# The basophil activation test by flow cytometry: recent developments in clinical studies, standardization and emerging perspectives

**DOI:** 10.1186/1476-7961-3-9

**Published:** 2005-06-30

**Authors:** Radhia Boumiza, Anne-Lise Debard, Guillaume Monneret

**Affiliations:** 1 Immunology Laboratory, Lyon-Sud University Hospital, Lyon, France; 2 Immunology Laboratory, Hôpital Neurologique, Lyon, France

**Keywords:** allergy, basophils, flow cytometry, CD63, CD203C, CRTH2

## Abstract

The diagnosis of immediate allergy is mainly based upon an evocative clinical history, positive skin tests (gold standard) and, if available, detection of specific IgE. In some complicated cases, functional *in vitro *tests are necessary. The general concept of those tests is to mimic *in vitro *the contact between allergens and circulating basophils. The first approach to basophil functional responses was the histamine release test but this has remained controversial due to insufficient sensitivity and specificity. During recent years an increasing number of studies have demonstrated that flow cytometry is a reliable tool for monitoring basophil activation upon allergen challenge by detecting surface expression of degranulation/activation markers (CD63 or CD203c). This article reviews the recent improvements to the basophil activation test made possible by flow cytometry, focusing on the use of anti-CRTH2/DP_2 _antibodies for basophil recognition. On the basis of a new triple staining protocol, the basophil activation test has become a standardized tool for *in vitro *diagnosis of immediate allergy. It is also suitable for pharmacological studies on non-purified human basophils. Multicenter studies are now required for its clinical assessment in large patient populations and to define the cut-off values for clinical decision-making.

## Introduction

Anaphylaxis consists of an immediate IgE-dependent reaction in response to allergens. Clinical symptoms are caused by an initial systemic histamine release by mast cells and basophils that may lead to shock with laryngeal edema, lower-airway obstruction and hypotension. The most frequent allergens involved in immediate allergy are found in peanuts, fish, bee and wasp venoms, drugs and latex [1,2]. The identification of responsible allergens remains a key step for practicing allergen avoidance and specific immunotherapy. The diagnosis is mainly based upon an evocative clinical history (including temporal association between symptoms and allergen exposure), positive skin tests, which remain the gold standard in this context and, if available, detection of specific IgE [3]. In most patients, these features allow both diagnosis and identification of the offending allergen. Nevertheless, skin testing is contraindicated in some patients with histories of life-threatening anaphylaxis, and discrepant results may be found between clinical assessment of the disease and biological results, especially for drug allergy. In these cases, functional *in vitro *tests are necessary. The general concept of those tests is to mimic *in vitro *the contact between allergens and the cells responsible for symptoms (*i.e,. *those possessing the ability to release histamine). Until recently, basophils were neglected and only considered to be circulating forms of mast cells of minor importance. Furthermore, basophils represent in peripheral blood less than 0.5 percent of total leukocytes, making their purification difficult in clinical laboratories. This lack of satisfactory *in vitro *protocols has clearly hampered research on basophils for many years [4]. Nevertheless, there is considerable recent evidence that basophils are clinically relevant. Indeed, they are now considered as equivalent to tissue mast cells cells since they play, by themselves, a pivotal role in the immediate allergic reaction [5-8]. Consequently, functional *in vitro *tests for allergic reactions are focused on circulating basophils. The first approach to basophil functional studies was the histamine release test. However, its clinical benefit has remained controversial due to insufficient sensitivity and specificity [3,9,10]. That is why several groups took advantage of flow cytometry to develop new tools for monitoring basophil activation upon allergen challenge by detecting surface expression of degranulation markers [11-13].

### Principle of basophil activation test by flow cytometry

**Figure 1:**
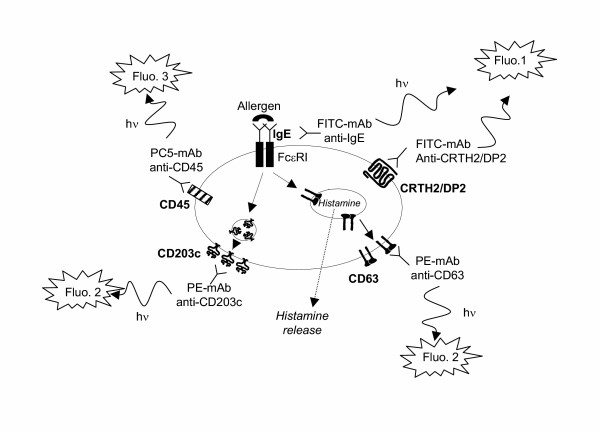
**Principle of the basophil activation test by flow cytometry (triple staining). **Basophils are identified on the basis of CD45 expression (fluorescence 3 / Phyco-Cyanine 5) and the presence of IgE or CRTH2/DP2 on their surface (fluorescence 1 / Fluorescein isothiocyanate). Resting basophils do not express CD63 (anchored in the basophilic granule) and weakly express CD203c. The cross-linking of two FcεRI (induced by an allergen or anti-IgE antibodies) provokes the histamine release (and as a consequence the CD63 expression) and the upregulation of CD203c. The rise in CD63 or CD203c expression (measured by fluorescence 2 / Phycoerythrin) before and after allergen challenge reflects thus the basophil activation / degranulation in response to an allergen.

As flow cytometry is a valuable tool for the analysis of many different cell types and can be used to identify specific populations of cells, even when present in low numbers, it seemed to be suitable for the study of allergen-induced basophil degranulation. Identification of cells was initially based both on CD45 expression, a common leukocyte antigen, and on the presence of IgE on the cell surface, since basophils express the high affinity receptor for IgE (FcεRI) [9,14]. In this gated population, cell activation upon allergen challenge was assessed by the expression of CD63 on the membrane [15,16]. CD63 is anchored in the basophilic granule membrane (which contains histamine) and its exposure to the outside of the cells reflects cell degranulation due to fusion between granules and plasma membranes (figure 1). Thus, CD63 expression has been proposed as a reliable means to monitor basophil activation [11-13]. Briefly, whole blood was incubated at 37°C with allergens for 15 minutes. The reaction was stopped on ice, followed by a 30-min staining with antibodies (figure 1). Finally, samples were lysed to eliminate red cells. Basophils expressing both CD45 and surface IgE were then examined for their CD63 expression. The threshold for positivity was determined with the use of a negative control (*i.e., *whole blood and vehicle without allergen). Results were considered positive when at least 2 sequential allergen dilutions induced greater than than 10% increases in CD63-positive basophils above control values. This kind of protocol has been validated for common allergens by several groups and has shown convincing results [17-24]. The technique has proven to be accessible, rapid (results in less than 1 hour) and requires small amount of blood (< 5 mL, even for assessing several allergens in the same experiment). In our hands, in allergy to muscle relaxants, the results were quite interesting, since we found the sensitivity of the CD63 test was similar to that for specific IgE detection and higher than the one for histamine release test [25]. This confirmed the value of performing the CD63 test rather than histamine release, which is furthermore costly in terms of both reagents and laboratory technician time. In accord with previous studies focusing on different allergens [17-21], this method showed excellent specificity. However, with respect to drug allergy, the main indication for this kind of test, three independent studies reported similar sensitivities ranging between 50 and 64 %, which is not sufficient for clinical usefulness [12,25,26]. In fact, this first approach relied on two important characteristics of basophils which were problematic: recognition through the expression of IgE on their surface (which is known to be highly variable from one patient to another) and the monitoring of their activation by detecting CD63 (which is also expressed to some extent by other activated leukocytes and by activated platelets that may adhere to basophils). This may explain why, when applied to drug allergy, these tests have remained somewhat disappointing in terms of sensitivities [12,25,26]. Consequently, we concluded that an activation marker that is more specific and/or sensitive than CD63 would be desirable.

### CD203c as a specific marker of activated basophils

**Figure 2:**
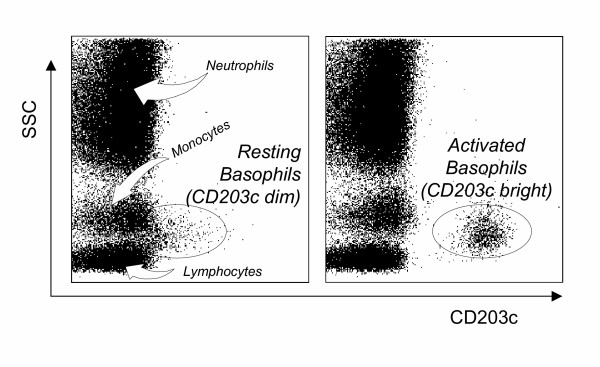
**CD203c expression in whole blood before and after basophil activation. **Ungated leukocytes are shown as a biparametric representation on the basis of side scatter characteristics (SSC, y-axis) and CD203c (x-axis). Left histogram depicts resting cells, basophils express low levels of CD203c (some of them are not distinguishable from lymphocytes and monocytes). Right histogram depicts cells after anti-IgE challenge, activated basophils are easily recognized on the basis of their high CD203c expression.

CD203c corresponds to a surface antigen expressed on human basophils recently recognized by the monoclonal antibody 97A6 [27]. This antigen, belonging to the type II transmembrane protein family, is a multifunctional ecto-enzyme called ectonucleotide pyrophosphatase phophodiesterase 3 (E-NPP3) [28] that catalyzes the cleavage of a number of molecules including deoxynucleotides and nucleotide sugars [29]. In addition, E-NPP3 contains a somatomedin B-like domain and a cell adhesive motif, but their potential functions remain totally unknown with respect to basophil physiology. Among leukocytes CD203c appears to be selectively expressed on the basophil/mastocytes lineage [27]. To date, no other cells from human peripheral blood have been reported to express this marker. Its expression on basophils is rapidly upregulated after stimulation with the appropriate allergen in patients sensitized to acarids or hymenoptera or after crosslinking of FcεRI with anti-IgE antibodies [28,30]. This suggests that CD203c up-regulation is more or less specific to the crosslinking of FcεRI (figure 2). Hence, as CD203c is rapidly upregulated after allergen challenge, it has been proposed as a new tool for allergy diagnosis [30-33]. We compared basophil activation tests using either CD63 or CD203c in the diagnosis of latex allergy [34] and found that the sensitivity was considerably higher with CD203c (75% compared to 50% with CD63). The improved sensitivity may be due to two factors. First, the recognition of basophils is better with CD203c. Indeed, the identification of basophils using prior protocols relied on a single IgE-labeling, although it is known that FcεRI expression can vary considerably on cell surfaces from one patient to another [35]. This may explain why in some cases basophils were difficult or impossible to identify. The second reason for the improved sensitivity with CD203c is due to its higher expression in activated basophils compared to CD63 in our experiments. In sensitized patients, basophils increased their CD203c levels up to 350 % above control values in response to allergens whereas the increase in CD63 was below 100 %. Similar results were obtained when expressing the results as the percentages of basophils that were CD203c- or CD63-positive. Even with the highest concentration of latex, the mean percentage of CD63-positive basophils was below 20 % while that of CD203c-positive basophils was 48 %, allowing a clear distinction between resting and activated basophils [34]. In conclusion, both easier gating and higher range of activation in response to allergen may contribute to an improvement in the basophil activation test when using CD203c rather than CD63. However, as very few studies concomitantly compared CD203c and CD63, this point remains to be confirmed by additional works dealing with various allergens. Bühring and colleagues in a recent report proposed to use both markers in the same test to increase sensitivity [32]. It is supported by recent evidence showing that CD63 and CD203c overexpression depend on different stimulatory pathways [36,37]. It is to note that some novel basophil-activation markers (CD13, CD107a, CD164) have been very recently identified [37]. They have to be further investigated in clinical studies either by their own or in combination with CD63 or CD203c.

### CRTH2/DP_2 _as a new marker for basophil recognition

**Figure 3:**
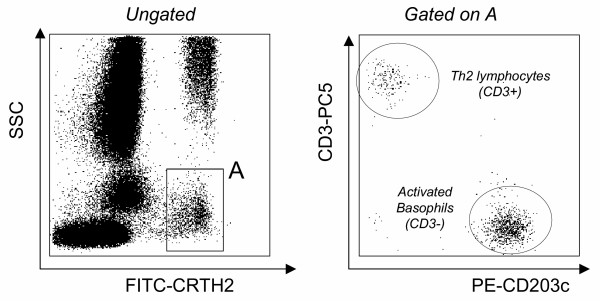
**Identification of CRTH2 expressing cells by flow cytometry. **Left histogram : ungated leukocytes biparametric representation on the basis of side scatter characteristics (SSC, Y axis) and FITC-CRTH2 (X axis). Two CRTH2 expressing cell populations are easily distinguishable: the one with high light scatterings corresponds to the eosinophil population; the second one (gating region: A) comprises Th2 lymphocytes and basophils. Right histogram: cells from the gating region (A) expressed on the basis of PE-CD203c (X axis) and PC5-CD3 (Y axis) characteristics. Th2 lymphocytes were readily separated from basophils based on their positive CD3 expression while activated basophils express high levels of CD203c without expressing CD3.

**Figure 4:**
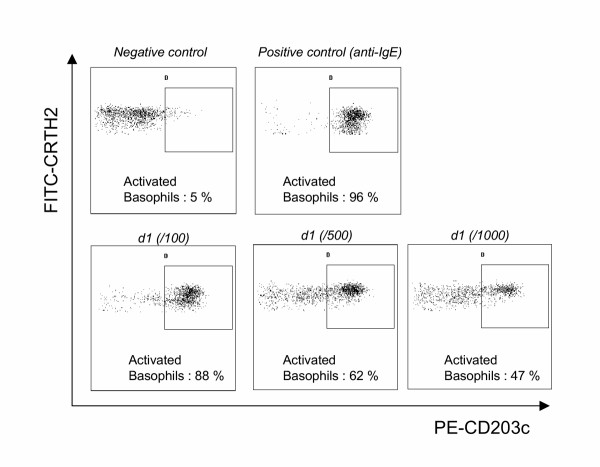
**Representative increased expression of CD203c after allergen challenge in a patient allergic to Dermatophagoïdes pteronyssinus (d1). **Gated CRTH2-positive basophils (after excluding Th2 lymphocytes as described in figure 3) are presented on the basis of CD203c-CRTH2 staining: before stimulation (negative control, upper left dot-plot), after anti-IgE challenge (positive control, upper right) and after allergen challenge at 3 different concentrations (dose-effect response, lower dot-plots). Activated basophils: percentage of basophils expressing CD203c.

Finally, the last drawback of the previously described protocols remained the use of an anti-IgE reagent to identify basophils. Because of its selective expression on cells associated with Th2 responses (Th2 lymphocytes, eosinophils and basophils), CRTH2 (chemoattractant receptor-homologous molecule expressed on Th2 cells)/DP_2 _has been proposed and validated as the most reliable tool for the detection of circulating human Th2 cells [38,39]. CRTH2 is also termed DP_2 _since it corresponds to the second receptor of prostaglandin D_2 _[40,41]. As CRTH2 is highly expressed on basophils, we hypothesized that it could improve the basophil activation test by facilitating basophil recognition. Consequently, we developed a new three-colour flow cytometric protocol (PE-CD203c / FITC-CRTH2 / PC5-CD3) for monitoring allergen-induced basophil activation. First results were encouraging: CRTH2 staining allowed CRTH2-expressing cells (eosinophils, basophils and Th2 lymphocytes) to easily be distinguished from other cells in samples of whole blood (figure 3). On the basis of light scattering, eosinophils were easily excluded from the analysis (figure 3). Basophils could then readily be distinguished from Th2 lymphocytes on the basis of CD3, staining, as this marker is not present on basophils (figure 3). Finally, on this gated population of basophils (low light scatterings, CRTH2+ and CD3-), modulation of CD203c after allergen challenge was monitored as described in the former protocol (figure 4). To validate this protocol, 18 subjects were included in a preliminary study [42]. Patients were allergic to either latex (k82) or *Dermatophagoïdes pteronyssinus *(d1), had a suggestive clinical history, positive skin test and/or specific IgE ≥ class III. Healthy donors, from our laboratory, were not known to be allergic and presented total IgE < 100 kU/L. In terms of clinical interpretation, sensitivity and specificity were 88% and 100%, respectively [40]. CRTH2 staining was an excellent means to identify basophils and we confirmed our earlier observations of a wide range of CD203c expression in response to allergen in tehse cells. In terms of basophil recovery, we compared our CRTH2-staining protocol with 2 others protocols using either anti-IgE or anti-CD123 (IL-3 receptor). In all patients and healthy individuals, we found more basophils (up to 50 % in certain patients) with the CRTH2-staining protocol, illustrating its superiority with respect to basophil recovery. To conclude, the easy recognition of basophils and the reliable assessment of their activation make this protocol the most reliable tool for investigating basophil activation by flow cytometry. It may constitute a critical step for the interlab standardization of this kind of test. Lastly, since CRTH2 is also a marker of Th2 cells and eosinophils, it may become a promising tool for flow cytometry, providing a direct overview of cells involved in "Th2 diseases" such as allergy.

### Perspectives in pharmacological studies

Until recently, due to the very low number of circulating basophils in humans, pharmacological studies on these cells were difficult to perform. This required large amount of blood and / or lengthy purification procedures that may induce nonspecific activation. By the use of flow cytometry, the effects of different compounds on basophils may be examined in unfractionated human blood cells. Recently, we have been able to demonstrate that among various eicosanoids, prostaglandin D_2 _was by far the most potent activator of basophils, inducing CD203c and CD11b elevation [37]. This response was mediated by the DP2 receptor / CRTH2 as it was shared by selective agonists of this receptor. As previously observed in eosinophils [39], the interaction of prostaglandin D_2 _with the DP_1 _receptor limited the activation of basophils by this prostaglandin. This suggested that the balance between DP_1 _and DP_2 _receptors may be crucial in determining the magnitude of basophil responses during allergic processes since prostaglandin D2 is known to be involved in allergic diseases and asthma. Using a similar approach, Heinemann et al. [43] examined the effects of various chemokines on human basophils and demonstrated a different pattern of chemokine receptor usage than those described for eosinophils and monocytes.

These studies illustrate that it is now possible to perform pharmacological and drug screening studies by flow cytometry. This approach could be very useful in assessing the possible risks of inducing anaphylactoid or pseudo-anaphylactoid reactions when developing new molecules. To this end, one important task for the future will be to extend these kinds of protocols to animal models although, to our knowledge, there is no available information on CD203c in animals and monoclonal antibodies directed against human CD203c do not cross-react with other species [32].

## Conclusion

After several improvements, the basophil activation test (using either CD203c or CD63 as activation marker) has become a robust and reliable test for *in vitro *investigations of immediate allergy, complementary to other existing *in vitro *tests. It is suitable for experimental and pharmacological studies as well as allergy diagnosis in clinical practice. There is now a crucial need for inter-laboratory standardization in clinical decision-making. Each allergen has to be assessed one by one to determine its optimal concentration (*i.e., *inducing maximal activation in vitro) as well as the definition of the threshold for positivity (using ROC analysis) since the use of an arbitrary cut-off value is likely not suitable for all allergens. The present challenge is to take advantage of the availability of improved methods to perform multicenter studies using a standardized protocol.

## Competing interests

The author(s) declare that they have no competing interests.

## Authors' contributions

RB participated as investigator and is the main author of the article.

ALD participated in drafting the manuscript.

GM was project leader and participated in the design of the different studies and drafting the manuscript.
